# Predictors of Poor Seizure Control in Children Managed at a Tertiary Care Hospital of Eastern Nepal

**Published:** 2016

**Authors:** Prakash POUDEL, Mohit CHITLANGIA, Rita POKHAREL

**Affiliations:** 1 Department of Pediatrics and Adolescent Medicine, B.P. Koirala Institute of Health Sciences, Dharan, Nepal; 2 Department of Psychiatric Nursing, B.P. Koirala Institute of Health Sciences, Dharan, Nepal

**Keywords:** Child, Seizures, Prognosis

## Abstract

**Objective:**

Various factors have been claimed to predict outcome of afebrile seizures in children. This study was aimed to find out the predictors of poor seizure control in children at a resource limited setting.

**Materials & Methods:**

This prospective study was done from July 1st, 2009 to January 31st, 2012 at B.P. Koirala Institute of Health Sciences, Nepal. Children (1 month-20 yr of age) with afebrile seizures presenting to pediatric neurology clinic were studied. Significant predictors on bivariate analysis were further analyzed with binary logistic model to find out the true predictors. Positive predictive values (PPVs) and negative predictive values (NPVs) for the true predictors were calculated.

**Results:**

Out of 256 patients (male: female ratio 3:2) with afebrile seizures followed up for median duration of 27 (IQR 12-50) months, seizure was poorly controlled in 20% patients. Three factors predicted poor seizure control. They were frequent (≥1 per month) seizures at onset (OR 12.76, 95% CI 1.44-112.73, PPV 25%, NPV 98%); remote symptomatic etiology (OR 3.56, 95% CI 1.04-12.17, PPV 36%, NPV 92%); and need of more than one anticonvulsant drug (polytherapy) (OR 12.83, 95% CI 5.50-29.9, PPV 56%, NPV 96%). The strongest predictor was need of polytherapy. When all three factors were present, PPV and NPV for prediction of poor seizure control were 70% and 90% respectively.

**Conclusion:**

Frequent seizures at onset, remote symptomatic etiology of seizure and need of polytherapy were associated with poor seizure control in children with afebrile seizures.

## Introduction

On the basis of available figures, from 1.5 to 5 per cent of any population have afebrile seizures at some time (1). Two unprovoked seizures greater than 24 h apart suggest the presence of an epileptic disorder (2). Epilepsy is common childhood morbidity and results from a variety of causes. Approximately 10-20% patients develop medically intractable epilepsy (3, 4). Early identification of patients who are likely to have poor seizure control is helpful in counseling patients and their families. This also helps in selecting patients fordetailed investigations, intensive treatments and for timely consideration of epilepsy surgery. In addition, determining risk factors for poor seizure control may stimulate further research on the causes and mechanisms underlying poor seizure control. Different factors are associated with poor seizure control in children. Commonly implicated factors in such studies are requirement of multiple antiepileptic drugs, remote symptomatic etiology, abnormal electroencephalogram (EEG), history of prior febrile convulsion, young age at onset of seizure, abnormal intelligence, frequent seizures at onset, neuroimaging abnormality, abnormal neurological examination findings, developmental delay, type of seizure, male gender and head trauma (5-18). Extensive investigations of an epileptic child using advanced modalities like Magnetic Resonance Imaging (MRI) may not be practical in resource limited areas due to issues related to availability and affordability. This study was carried out to find out the simple clinical and laboratory factors that predict poor seizure control in children at a resource limited setting.

## Materials & Methods

This prospective hospital based study was conducted at a university teaching hospital in eastern Nepal. This multispecialty tertiary care hospital provides medical services for children with seizures referred from whole eastern part of Nepal through its pediatric neurology clinic. All patients (1 month to 20 years of age) attending to pediatric neurology clinic with history of at least one definite unprovoked afebrile seizure were enrolled using consecutive sampling method into the study from July 1st, 2009 to August 31st, 2011. They were followed up till January 31st, 2012. Neonates with seizures, patients with febrile convulsion and acute symptomatic seizures were excluded from the study. Ethical clearance was obtained from institutional Ethical Review Board. Written informed assents to participate in the study were obtained from parents. At first visit, information about patient’s sociodemographic profile, history, possible risk factors and other important parameters were recorded in predesigned data collection sheet. Diagnoses of seizure and epilepsy were made depending upon clinical description and history given by parents or eyewitness. Physicalexamination was done and findings were recorded. Relevant investigations like computed tomography (CT) scan and EEG were advised. EEG was considered as abnormal when there was epileptiform discharge or slow wave abnormality. Seizures were classified using international classification of epileptic seizures proposed by International League against Epilepsy (ILAE) in 1981(19). Patients were treated and followed up in Pediatric Neurology Clinic. The follow-ups were done every 3 months for patients with well controlled seizure and more frequently on need basis for patients with poorly controlled seizures. During follow-up, details of compliance, complications of antiepileptic drugs, status of seizure control and modification in treatment were evaluated and recorded. Duration of follow-up was calculated by adding retrospective follow-up duration (time from first seizure to first medical attention at pediatric neurology clinic) as obtained from history and prospective follow-up duration (time from first presentation at the clinic to latest visit before February 2012). Data were entered and screened for error in MS Excel. The analysis was done using SPSS 16.0 software (Chicago, IL, USA). Seizure was considered to be under good control when a patient remained seizure free either for at least two months or for two times the length of usual pretreatment interictal interval, whichever was longer. Patients with uncertain final seizure control status because of insufficient follow-up duration or loss to follow-up were excluded from outcome analysis. The factors that could potentially affect the final outcome were compared between the groups of children who achieved and who did not achieve good seizure control. The factors that were significantly different between the groups on bivariate analysis using the Chi square test (P<0.05) were further analyzed with binary logistic regression model to find out the true predictors. Fitness of model was tested using Hosmer and Lemeshow test. Positive predictive value (PPV), negative predictive value (NPV), odds ratio (OR), sensitivity and specificity for each predictor and combination of the predictors were calculated. P value of 0.05 was taken as cut off for statistical significance. Based on available literature, many predictors were presumed to have potential predictive value. Thepredictors thus considered for analysis were - Age of onset below 5 yr, frequent seizures at onset (≥1 seizures per month), CT scan abnormality, EEG abnormality, abnormal neurological examination finding, family history of seizure, history of status epilepticus, remote symptomatic etiology (underlying chronic brain pathology) of seizure, need of polytherapy (more than one antiepileptic drugs) and clinically focal onset of seizure. 

## Results

Out of 308 enrolled patients, 52 were excluded due to inadequate data and/or inadequate duration of follow-up. Final analysis was done on 256 patients.

**Clinical characteristics**

There were 151 male and 105 female patients. Male: female ratio was 3:2. Median age at first seizure was 36 (Interquartile range (IQR) 12 - 96) months. Median age at presentation was 84 (IQR 36 - 144) months. Median Follow-up duration was 27 (IQR 12 - 50) months. Table 1 shows clinical characteristics of patients. Bivariate analysis was used to compare the potential predicting factors between the groups of children with and without good seizure control (Table 2). Chi square test was used to test the statistical significance of the difference. The factors that were significantly different between the groups on bivariate analysis were further entered into multivariate model and analyzed with binary logistic regression method. Fitness of model was tested with Hosmer and Lemeshow test (Chi square 8.793, Significance 0.268). Accuracy of model was 86% as shown by classification table. Table 3 shows results of binary logistic regression analysis for detection of true predictors. Table 4 shows predictive and diagnostic accuracy of each and combination of the predictors in terms of PPV, NPV, sensitivity and specificity.

## Discussion

Studies over the last several years have significantly improved current knowledge of the etiology, incidence and prognosis of epileptic seizures in children. Predictors of seizure control in epileptic children have been examined in many studies. Results as well as definitionof poor seizure control are not uniform among these studies. In a study to define intractable epilepsy, a child had to have uncontrolled seizures that occurred with an average frequency of at least 1 per month for a period of at least 2 yr (5). Malik et al. and Kwong et al. also used the almost similar criteria for seizure intractability (6, 7). Though this definition seems appropriate, it requires a long and strict follow-up that may be less practical for short term studies. Therefore, we have defined good seizure control when patient remained seizure free for either at least two months or for two times the length of usual pretreatment interictal interval, whichever was longer. Chawla et al. defined poor seizure control in children who had more than one seizure per month over at least 6 months (8). Many studies have found various factors as predictors of poor outcome of seizure (Table 5). Up to 70% of childhood epilepsies will respond to the first or second antiepileptic drug. If two appropriate antiepileptic drugs have failed independently as monotherapy, the chance of further monotherapy controlling seizures is very low and combination therapy should be considered (20). In a large German study done in childhood seizure, 74.4% children were successfully managed with monotherapy (21). In our study, 71.5% patients responded to monotherapy. On logistic regression analysis, strongest predicting factor in our study was need of polytherapy (more than one antiepileptic drug) (OR 20, PPV 56% and NPV 94%). Failure of first anticonvulsant drug was associated with poor outcome (16). Treatment with more than 3 antiepileptic drugs was a significant negative outcome predictor in one of the studies carried out in Germany in children with difficult to treat epilepsy (17). Remote symptomatic etiology of seizure was the next important factor predicting poor outcome in our study (OR 6.1, PPV 36% and NPV 92%). This could be because of underlying chronic brain pathology acting as persistent focus for seizure. Remote symptomatic etiology was significantly negative outcome predictor by other studies also (5, 7-12). Etiology was predictive not only of seizure outcome but also of mortality in childhood-onset epilepsy (11). In contrast, cryptogenic etiology predicted poor outcome in a study (6). Third factor predicting poor seizure control in our study was frequency of seizure at onset. Only approximately 30% to 40% of patients with a first seizure will have a second unprovoked seizure (22). Therefore, outlook of patients with single seizure is expected to be better than those with recurrent seizures. Long term outcome was good when there was single seizure without recurrence (13). In our study, if seizure was frequent (≥ 1 seizure per month) at onset, seizure control was poor (OR 17, PPV 25% and NPV 98%). Frequency of seizure is probably related to severity of underlying brain pathology and dysfunction, thus determining the outcome. More than one seizure per month was associated with poor seizure control (14). More than three seizures in the second 6 months after starting treatment predicted intractable epilepsy (7). Recurrence of seizure in the first 6 to 12 months of treatment (hazard ratio 70) predicted poor outcome (15). Early seizure frequency predicted longterm seizure control during antiepileptic drug treatment (11). It appears that initial seizure frequency before treatment as well as shortly after treatment is predictive of outcome.Younger age at onset of seizure has been found to be associated with poor outcome by some studies. But the cut off ages taken by those studies were different. Age at onset of seizure less than 1 year (6, 8, 12), less than 3 years (9, 10), less than 5 years (18) and less than 14 years (14) predicted poor outcome in those studies. Younger age at onset of seizure was associated with poorer outcome (5). In our study, cut off age was taken as 5 years. Although seizure onset below the age of 5 years was significant predictor of poor outcome on bivariate analysis, the significance was lost on logistic regression analysis. One study done in Thai children also failed to show relationship of age at onset with outcome of seizure (23). The type of seizure predicted outcome. Focal (14), myoclonic (6, 8), neonatal (6), infantile spasm (5, 8) and mixed seizure types (15) have been found to be associated with poor outcome in children. We classified the seizure types based upon clinical semiology. We checked the relationship between focal seizure and the outcome. We failed to establish any associationbetween focal onset of seizure and the final seizure control status. It could be because we did not strictly follow electro-clinical classification, but relied upon the clinical semiology to categorize seizure onset into generalized and focal. The classification of seizure type is often difficult and focal onset seizures are frequently underreported. Numerous so-called generalized seizures in various studies, including our study, are probably secondarily generalized. The recognition of a focal onset depends upon the skill of the investigator and the range of investigations. In day to day practice, clinicians frequently classify seizure on the basis of seizure observation or description only (23). This is truer in resource limited settings where investigations are costlier and not easily available as compared to that in resource rich countries.Other factors like abnormal EEG (6,9,10,13), abnormal CT scan/neuroimaging (8,14,16), developmental delay (7,14,15), abnormal intelligence(15,18), prior febrile convulsion (9,10,14), male gender (6) and head trauma (6) have been predictors of poor seizure control by various studies. Abnormality in CT scan, abnormal EEG, abnormal neurological examination findings, family history of seizure, prior febrile convulsion and prior status epilepticus were not associated with final seizure control status in patients of our study. Prospective design and strong statistical methods are the strengths of our study. MRI is preferred to CT scan in investigation of epilepsy. Due to limitation of resources, we could not obtain MRI in our patients. Therefore, symptomatic etiologies might have been missed in some of our patients. Higher proportion of EEG abnormality in our study reflects over-interpretation. The EEG may show paroxysmal activity or background changes in up to 32% of normal children that could be misread as abnormal (25). We had to exclude 52 patients from final analysis because they were lost to follow-up. We followed clinical semiology for seizure classification. Follow-up duration was also relatively shorter and not uniform in all patients. These are some of the limitations of our study. This study tries to predict seizure outcome in children managed at a low income country depending upon simple risk factors. These factors can be easily detected and used in any resource constrained setting for counseling parents and to speculate the outcome. However, the prediction of the outcome can be made further accurate by finding out additional predictors by more sophisticated neuroimaging, genetic and metabolic investigations, especially in rich countries where affordability and availability of such investigations are not the issues. In conclusion, seizures are likely to be poorly controlled in children when the initial seizure frequency is high (≥1 per month), or the etiology of seizure is remote symptomatic, or when seizure does not respond to one anticonvulsant drug. Need of polytherapy is the strongest predictor of poor outcome. As the number of predictors present in a child increase, accuracy of prediction increases. This study is not adding new information regarding predictors per se because the predicting factors identified by this study have been previously recognized by other similar studies in many areas of the world (5- 17). However, this study is of importance because it adds substantial evidence to the findings of those studies and provides new information regarding accuracy and strength of prediction.

**Table 1:** Clinical Characteristics of Children with Afebrile Seizure

Characteristics	Number	Percent
**Frequent seizures at onset**	204	80
**History of status epilepticus**	67	26
**Prior febrile convulsion**	20	8
**Family history of seizure**	34	13
**Abnormal neurological examination**	59	23
**Abnormal EEG** *	189	74
**Abnormal CT ** ^†^ **scan**	78	30.5
**Remote symptomatic etiology**	112	43.8
**Response to single anticonvulsant drug**	183	71.5
**Clinical seizure type**		
** Generalized **	200	78
** Focal onset**	38	15
** Mixed and unclassified**	18	7
**Poor seizure control**	52	20

* EEG, Electroencephalogram;

† CT, Computerized Tomography

**Table 2:** Comparison of Predicting Factors between Children who did Not Achieve (Poor Control Group) and who Achieved (Good Control Group) Good Seizure Control

**Potential predictors**	**Poor control group** **n** † ** = 52**	**Good control group** **n = 204**	***P***
Seizure onset below 5 years of age, n (%)	41 (79)	111 (54)	0.001*
Frequent seizures at onset, n (%)	51 (98)	153 (75)	<0.001*
Abnormal neurological examination, n (%)	29 (56)	30 (15)	<0.001*
Remote symptomatic etiology, n (%)	40 (77)	72 (35)	<0.001*
Requirement of polytherapy, n (%)	41 (79)	32 (16)	<0.001*
Abnormal CT‡ scan, n (%)	28 (54)	50 (25)	<0.001*
History of status epilepticus, n (%)	11 (21)	56 (28)	0.356
Abnormal EEG§, n (%)	42 (81)	147 (72)	0.202
Family history of seizure, n (%)	5 (10)	29 (14)	0.383
History of prior febrile convulsion, n (%)	2 (4)	18 (9)	0.233
Focal onset of seizure, n (%)	8 (15)	30 (15)	0.902

‡ CT, Computerized

† n, Number;

* Statistically significant (*P*<0.05); Tomography;

§ EEG, Electroencephalogram

**Table 3:** Binary Logistic Regression Results for Detection of Predictors

**Predictors **	**Significance **	**Adjusted Odds Ratio **	**95% CI ** ^†^
Frequent seizure at onset	0.022*	12.76	1.44 - 112.73
Remote symptomatic etiology of seizure	0.043*	3.56	1.04 - 12.17
Need of polytherapy	0.000*	12.83	5.50 - 29.91
Abnormal CT‡	0.63	1.3	0.44 - 3.78
Abnormal neurological examination	0.47	1.41	0.53 - 3.73
Seizure onset below 5 years of age	0.118	2.07	0.83 - 5.19

* Statistically significant (*P*<0.05);

† CI, Confidence Interval;

‡ CT, Computerized Tomography

**Table 4:** Diagnostic and Predictive Accuracy of Predictors (%)

**Predictor **	**Sensitivity **	**Specificity **	**Odds Ratio (95 CI** * **) **	**PPV** †	**NPV** ‡
Frequent seizure at onset (A)	98	25	17 (2.3-126.2)	25	98
Remote symptomatic etiology (B)	77	65	6.1 (3.1-12.4)	36	92
Need of polytherapy (C)	79	84	20.1 (9.3-40.1)	56	94
A+B	75	73	7.9 (3.9-15.9)	41	92
B+C	60	92	17.3 (8.2-36.8)	66	90
A+C	77	86	20.9 (9.8-41.8)	59	94
A+B+C	58	94	20.1 (9.1-44)	70	90

* CI, Confidence Interval;

† PPV,Positive Predictive Value;

‡ NPV, Negative Predictive Value.

**Table 5:** Factors Predicting Poor Seizure Control as Found in Various Studies

**Predictors**	**Referencs**
Remote symptomatic etiology	Berg et al.**(**5**),**Kwong et al. **(**7**),**Chawla et al.**(**8**),**Shinnar et al**. (**9**),**Shinnar et al. **(**10**),**Sillanpaa et al. **(**11**),**Casetta et al. **(**12**)**
High initial seizure frequency	Malik et al. **(**6**),**Kwong et al. **(**7**),** Sillanpaa et al. **(**11**),**Casetta et al. **(**12**),**Stroink et al. **(**13**),**Tripathi et al. **(**14**),**Oskoui et al. **(**15**)**
Need of polytherapy	Tripathi et al. **(**14**),**Lohani et al. **(**16**),**Beume et al. **(**17**)**
Young age at onset	Berg et al. **(**5**),**Malik et al. **(**6**),**Chawla et al. **(**8**),**Shinnar et al**. (**9**),**Shinnar et al. **(**10**),**Casetta et al. **(**12**),**Tripathi et al. **(**14**),**Bouma et al. **(**18**)**
Abnormal electroencephalogram	Malik et al. **(**6**),**Shinnar et al**. (**9**),**Shinnar et al. **(**10**),**Stroink et al. **(**13**)**
Abnormal neuroimaging	Tripathi et al. **(**14**)**
Abnormal neurological examination	Chawla et al. **(**8**),**Lohani et al. **(**16**)**
Abnormal development	Kwong et al. **(**7**),**Tripathi et al. **(**14**),**Oskoui et al. **(**15**)**
Abnormal intelligence	Oskoui et al. **(**15**),**Bouma et al. **(**18**)**
Seizure type	Berg et al. **(**5**),** Malik et al. **(**6**),**Chawla et al. **(**8**),**Tripathi et al. **(**14**),**Oskoui et al. **(**15**)**
Prior febrile convulsion	Shinnar et al**. (**9**),**Shinnar et al. **(**10**),**Tripathi et al. **(**14**)**
Male gender	Malik et al. **(**6**)**
Head trauma	Malik et al. **(**6**)**
Cryptogenic etiology	Malik et al. **(**6**)**
